# Determining the concentration of golden sea cucumber (*Stichopus herrmanni*) extract gel in oral traumatic ulcers

**DOI:** 10.1016/j.jtumed.2026.06.009

**Published:** 2026-07-07

**Authors:** Pratiwi Soesilawati, Yuliati, Dian W. Damaiyanti, Muhammad A.B. Firdauzy, Michael S.C. Panggabean, Muhammad Ramadhan, Anindita R. Larasati, Ira Arundina, Clara N. Dewiyanti, Rafael Gerrard, Evelyn N. Prasetyo, Noraini Ahmad

**Affiliations:** a Department of Basic Dental Sciences and Public Health, Faculty of Dental Medicine, Universitas Airlangga, Surabaya, Indonesia; b Department of Innovation and Research, Universitas Airlangga Hospital, Surabaya, Indonesia; c Faculty of Dental Medicine, Universitas Hang Tuah, Surabaya, Indonesia; d Immunology Program, Postgraduate School, Universitas Airlangga, Surabaya, Indonesia; e Department of Periodontology, Periodontology Specialist Resident, Faculty of Dental Medicine, Universitas Airlangga, Surabaya, Indonesia; f Postgraduate School, Faculty of Dental Medicine, Universitas Airlangga, Surabaya, Indonesia; g Undergraduate Student, Faculty of Dental Medicine, Universitas Airlangga, Surabaya, Indonesia; h Department of Chemistry, Faculty of Science, University Malaya, Kuala Lumpur, Malaysia; i Doctoral Program, Faculty of Medicine, Universitas Airlangga, Surabaya, Indonesia

**Keywords:** خيار البحر الذهبي, *Stichopus herrmanni*, القرحة الرضّية؛ الخلايا البلعمية, الخلايا البدينة, تكوّن الأوعية الدموية, إعادة تكوّن الظهارة, Angiogenesis, Macrophages, Mast cells, Reepithelialization, *Stichopus herrmanni*, Traumatic ulcer

## Abstract

**Objective:**

Traumatic oral ulcers are common lesions that may interfere with oral function and quality of life. Golden sea cucumber (*Stichopus herrmanni*) contains various bioactive compounds with potential wound healing properties. This study aimed to evaluate the effects of different concentrations of golden sea cucumber extract gel on traumatic oral ulcer healing in Wistar rats.

**Methods:**

Twenty male Wistar rats were randomly divided into four groups: control, 20%, 40%, and 80% golden sea cucumber extract gel groups. Traumatic ulcers were induced on the lower labial mucosa using a heated burnisher. The treatment groups received topical application of the extract gel once daily. Histopathological evaluations were performed on days 4 and 7 post-ulcer induction. The observed parameters included macrophage count, mast cell count, angiogenesis, collagen density, and re-epithelialization. Statistical analysis was performed using one-way ANOVA followed by Tukey's post hoc test, with p < 0.05 considered statistically significant.

**Results:**

The 40% concentration group demonstrated higher angiogenesis compared with the other groups during the early healing phase. Meanwhile, the 80% concentration group showed greater macrophages, epithelial thickness, and collagen density during the proliferative phase. Significant differences were observed among treatment groups in several histological parameters (p < 0.05), indicating enhanced wound healing activity following golden sea cucumber extract administration.

**Conclusion:**

*Stichopus herrmanni* extract gel accelerated traumatic oral ulcer healing by modulating inflammation, promoting angiogenesis, enhancing re-epithelialization, and increasing collagen deposition. Different concentrations exhibited distinct therapeutic effects at different stages of oral wound healing, suggesting its potential as a natural adjunctive therapy for traumatic oral ulcers.

## الملخص

**أهداف البحث:**

تُعد القرحات الفموية الرضّية من الآفات الشائعة التي قد تؤثر في وظائف الفم وجودة الحياة. ويحتوي خيار البحر الذهبي، المعروف علميًا باسم *Stichopus herrmanni*، على مركبات حيوية فعالة متعددة قد تمتلك خصائص داعمة لالتئام الجروح. هدفت هذه الدراسة إلى تقييم تأثير هلام مستخلص خيار البحر الذهبي بتراكيز مختلفة في التئام القرحات الفموية الرضّية لدى جرذان ويستار.

**طرق البحث:**

وُزعت عشرون جرذًا من ذكور ويستار عشوائيًا إلى أربع مجموعات: مجموعة ضابطة، ومجموعات عولجت بهلام مستخلص خيار البحر الذهبي بتراكيز 20% و40% و80%. وأُحدثت القرحات الرضّية في مخاطية الشفة السفلية باستخدام أداة صقل مسخنة. وتلقت مجموعات العلاج تطبيقًا موضعيًا للهلام مرة واحدة يوميًا. وأُجري التقييم النسيجي المرضي في اليومين الرابع والسابع بعد إحداث القرحة. وشملت المتغيرات المقاسة عدد الخلايا البلعمية، وعدد الخلايا البدينة، وتكوّن الأوعية الدموية، وكثافة الكولاجين، وإعادة تكوّن الظهارة. وأُجريت التحليلات الإحصائية باستخدام تحليل التباين أحادي الاتجاه، يليه اختبار توكي للمقارنات البعدية، واعتُبرت القيمة الاحتمالية الأقل من 0.05 ذات دلالة إحصائية.

**النتائج:**

كان تكوّن الأوعية الدموية أعلى خلال المرحلة المبكرة من الالتئام عند استخدام تركيز 40% مقارنةً بالعلاجات الأخرى. كما كان عدد الخلايا البلعمية، وسماكة الظهارة، وكثافة الكولاجين أعلى خلال مرحلة التكاثر عند استخدام تركيز 80%. ولوحظت فروق ذات دلالة إحصائية بين العلاجات في عدد من المؤشرات النسيجية المرضية (p < 0.05)، مما يشير إلى تعزيز نشاط التئام الجروح بعد استخدام مستخلص خيار البحر الذهبي.

**الاستنتاجات:**

سرّع هلام مستخلص خيار البحر الذهبي التئام القرحات الفموية الرضّية من خلال تنظيم الاستجابة الالتهابية، وتعزيز تكوّن الأوعية الدموية، وتحسين إعادة تكوّن الظهارة، وزيادة ترسيب الكولاجين. وأظهرت تراكيز الهلام المختلفة تأثيرات علاجية متباينة خلال مراحل التئام الجروح الفموية، مما يشير إلى إمكان استخدامه علاجًا مساعدًا طبيعيًا للقرحات الفموية الرضّية.

## Introduction

Traumatic ulcers in the oral cavity are common lesions caused by physical injury, such as sharp tooth cusps, mechanical irritation from dental appliances, or accidently biting oral tissues, leading to epithelial disruption and exposing the underlying connective tissue. Clinically, traumatic ulcers present as painful defects with erythematous borders and a necrotic pseudomembrane, causing discomfort.^1^ Management of traumatic ulcers is typically supportive, involving removal of causative factors and applying topical agents such as corticosteroids or anesthetics, but healing may be delayed in some patients due to persistent inflammation or suboptimal tissue regeneration.^2^ Delayed healing increases the risk of secondary infection and can prolong patient morbidity, highlighting the limitations of current therapies that focus primarily on symptom control rather than accelerating the biological healing processes. Despite the availability of a range of clinical treatments, interventions are still required that promote faster and more effective mucosal repair. Recent research has shifted attention toward adjunctive therapies that modulate healing at the cellular level to achieve better outcomes.^3^ Therefore, understanding the underlying mechanisms involved is essential for developing more targeted therapeutic strategies.

Wound healing in the oral mucosa involves a coordinated sequence of biological events that restore tissue integrity after injury. The process is typically divided into four overlapping phases: hemostasis, inflammation, proliferation, and remodeling. During hemostasis, blood clot formation provides a provisional matrix that supports cell migration, which is followed by the inflammatory phase where neutrophils and macrophages clear debris and orchestrate cytokine signaling.^3^ The proliferative phase involves angiogenesis, fibroblast proliferation, collagen synthesis, and re-epithelialization, which are all critical for rebuilding the soft tissue structure. Finally, in the remodeling phase, collagen is reorganized and the tissue tensile strength increases. Oral mucosal wounds often heal faster and with less scarring compared with skin wounds, partly due to differences in cellular responses and the unique oral microenvironment.^4^ However, impaired regulation of these phases can delay closure and functional recovery.^5^

Natural products have gained significant interest for wound management because their bioactive properties can help to modulate inflammation and tissue regeneration. In particular, bioactive compounds from marine organisms such as sea cucumbers have attracted attention due to their therapeutic potential. Sea cucumbers are rich in glycosaminoglycans, saponins, collagen, and peptides with anti-inflammatory, antioxidant, and wound-healing activities.^6^ In vivo and clinical studies have shown that extracts from various sea cucumber species can enhance tissue repair by regulating inflammatory responses, promoting fibroblast proliferation, and facilitating angiogenesis. In particular, the golden sea cucumber (*Stichopus herrmanni Semper*), which is abundant in Indonesia, contains high amounts of glycosaminoglycans and collagen that could support mucosal healing by stimulating key wound-healing pathways and growth factors.^7^ These findings suggest a potential role for *Stichopus herrmanni* extracts as alternative therapeutic agents in oral wound care.

Despite growing evidence regarding the general wound-healing effects of sea cucumber extracts, scientific data remain limited about the effects of freeze-dried *Stichopus herrmanni* extracts on macrophage expression and mucosal repair in experimental traumatic ulcers. Previous studies focused primarily on endpoints such as lymphocyte counts, vascular endothelial growth factor expression, and collagen deposition, and thus comprehensive evaluations of specific immune cell modulation in the healing cascade are lacking. Therefore, the aim of this study was to evaluate the effect of freeze-dried *Stichopus herrmanni* extract on macrophage expression in traumatic ulcers of the labial mucosa in Wistar rats, thereby providing new insights into its therapeutic potential.

## Materials and Methods

### Study design and experimental animals

This study employed a randomized post-test only control group design with a laboratory experimental approach. In total, 20 healthy male Wistar rats (*Rattus norvegicus*), weighing 200–300 g and aged 8–16 weeks, were used as experimental subjects. All animals had normal lower labial mucosa without visible pathological abnormalities prior to the experiment. The animals were randomly allocated to four groups (n = 5 per group): a control group without treatment, and three treatment groups that received *Stichopus herrmanni* extract gel at concentrations of 20%, 40%, and 80%.

### Preparation of Stichopus herrmanni extract gel

*Stichopus herrmanni* individuals were washed thoroughly under running water, and the internal organs were removed. The body tissues were cut longitudinally, dried using filter paper, and homogenized until a smooth consistency was obtained. Fifty grams of homogenized tissue were mixed with 100 mL of distilled water in a 250 mL Erlenmeyer flask and shaken in a water-bath shaker at 80 rpm and 30 °C for 4 h. The mixture was then centrifuged at 3000 rpm for 20 min, and the supernatant was collected. The extract was subsequently freeze dried using a freeze dryer (Heto FD3, ID 87164) to obtain a powdered extract, which was stored in a sterile container at 4 °C until use.

The gel base was prepared using polyethylene glycol (PEG) 400 and PEG 4000 at a ratio of 1:2. The mixture was melted in an oven at 150 °C for 1 h and stirred until homogeneous, then allowed to cool. The freeze-dried *Stichopus herrmanni* extract was incorporated into the gel base to obtain concentrations of 20%, 40%, and 80%, and mixed thoroughly until a uniform gel consistency was achieved. The prepared gels were stored in airtight containers. All extraction and gel preparation procedures were performed at Surabaya Industrial Research and Consultation Laboratory (Surabaya, Indonesia).

### Induction of traumatic ulcer and treatment procedure

The experimental animals were anesthetized by using inhalation anesthesia with 10% ether administered via a cloth until the animals were sedated and showed abdominal breathing. The lower labial mucosa was disinfected using 0.12% chlorhexidine digluconate. Traumatic ulcers were induced by applying a heated egg-shaped burnisher (No. 4) with a diameter of 2 mm to the lower labial mucosa for 1 s after heating for 1 min.

On the third day following ulcer induction, the treatment groups received topical application of *Stichopus herrmanni* extract gel at concentrations of 20%, 40%, or 80%, where a dose of 0.1 mg was applied once daily to the ulcer area until euthanasia. The control group did not receive extract application. The experimental animals were divided into two observation subgroups corresponding to days 4 and 7 post-ulcer induction. Day 4 was selected to evaluate the early inflammatory phase of wound healing, particularly cellular inflammatory responses such as macrophage and mast cell activity.^3^ In addition, day 7 was selected to represent the early proliferative phase, characterized by angiogenesis, collagen deposition, and re-epithelialization during oral mucosal healing.^4^ Euthanasia was performed on days 4 and 7 post-ulcer induction, and tissue samples were collected from the lower labial mucosa, including both ulcerated and surrounding normal tissue.

### Histological preparation

Excised tissue specimens were fixed in buffered formalin solution for 12–18 h, before dehydration using a graded ethanol series, clearing with xylol, and embedding in paraffin blocks. The paraffin-embedded tissues were sectioned serially at a thickness of 4–6 μm using a rotary microtome. The tissue sections were mounted on glass slides and stained with hematoxylin and eosin (HE) to evaluate the macrophage count, mast cell count, angiogenesis, and re-epithelialization. Masson's trichrome staining was performed to assess collagen fiber formation. Histological processing was carried out at Dr. Soetomo General Hospital (Surabaya, Indonesia).

### Histological evaluation

HE-stained sections were examined under a light microscope (Nikon H600L, Nikon Corporation, Tokyo, Japan) and an inverted microscope (Olympus IX71, Olympus Corporation, Tokyo, Japan) at 200× magnification. Observations were conducted at the ulcer repair site across five random fields of view with a diameter of 70 μm. Macrophages were identified based on their oval or kidney-shaped bluish-purple nuclei with reddish cytoplasm, mast cells according to their granule-containing cytoplasm stained blue, and angiogenesis by counting newly formed capillaries characterized by clear lumens containing red blood cells. Re-epithelialization was assessed by measuring the epithelial thickness.^8^

The collagen fiber density was evaluated in sections stained with Masson's trichrome at 100 × magnification. Collagen fibers were observed as blue-stained elongated structures. The collagen density was assessed semi-quantitatively by three independent histology experts using the following scoring system: 0 (no collagen fibers), +1 (low density, <10%), +2 (moderate density, 10–50%), +3 (high density, 50–90%), and +4 (very high density, 90–100%).^7^^,^^8^

### Statistical analysis

Statistical analysis was performed using SPSS software version 26.0 (IBM Corp., Armonk, NY, USA). Data normality was assessed using the Shapiro–Wilk test. Differences among groups were assessed using one-way analysis of variance followed by Tukey's post hoc test for multiple comparisons. A *p*-value less than 0.05 was considered to indicate a statistically significant difference.

## Results

### Mast cells

**Figure 1:**
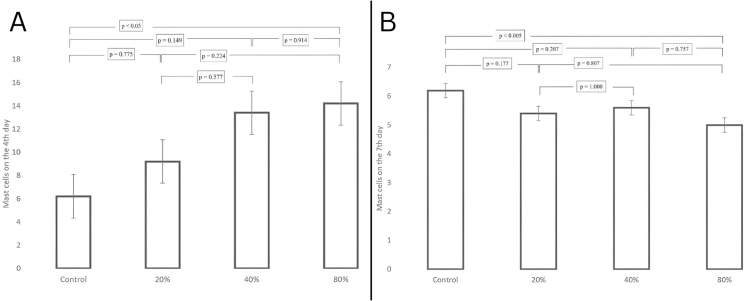
Effect of *Stichopus herrmanni* on mast cell counts in traumatic oral ulcers. (A) Mean number of mast cells in traumatic oral ulcers on day 4 following topical application of *Stichopus herrmanni* extract gel at concentrations of 20%, 40%, and 80% compared with the control group. (B) Mean number of mast cells in traumatic oral ulcers on day 7 after treatment with the same concentrations of *Stichopus herrmanni* extract gel.

**Figure 2:**
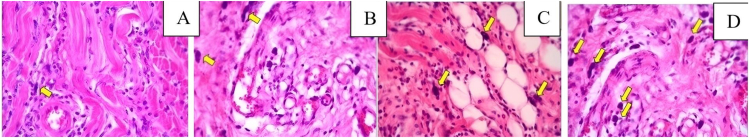
Histology of mast cells on day 4 (HE; 200×). (A) Control group, (B) 20% extract treatment group, (C) 40% extract treatment group, and (D) 80% extract treatment group.

**Figure 3:**
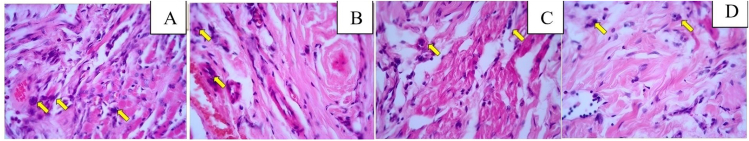
Histology of mast cells (yellow arrow) on day 7 for traumatic ulcers in Wistar rats (HE; 200×). (A) Control group, (B) 20% extract treatment group, (C) 40% extract treatment group, and (D) 80% extract treatment group.

The mast cell counts on days 4 and 7 are presented in Figure 1, and representative histological findings are shown in Figure 2 (day 4) and 3 (day 7). On day 4, the number of mast cells was higher in all of the extract-treated groups compared with the control group, with the highest count observed in the 80% extract group. Statistical analysis detected a significant difference only between the control group and the 80% extract group on day 4 (*p* < 0.05), whereas no significant differences were found between the control group and the 20% or 40% extract groups (*p* > 0.05). According to histological analyses, Figure 2 shows that mast cells were observed as oval to round cells with basophilic cytoplasmic granules, which were sparsely distributed in the control group and more frequently observed in the 80% extract group. On day 7, the mast cell counts decreased in all extract-treated groups, whereas the control group had a relatively similar number compared with that on day 4. As shown in Figure 3, histological observations detected fewer mast cells within the connective tissue in the extract-treated groups, particularly in the 80% group, compared with day 4. Statistical analyses detected a significant difference only between the control group and the 80% extract group on day 4 (*p* < 0.05).

### Macrophages

**Figure 4:**
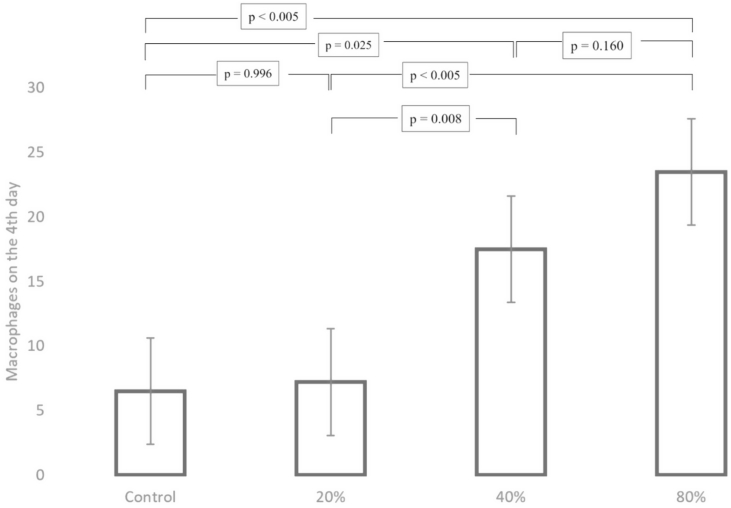
Effect of *Stichopus herrmanni* extract on macrophage counts on day 4.

**Figure 5:**
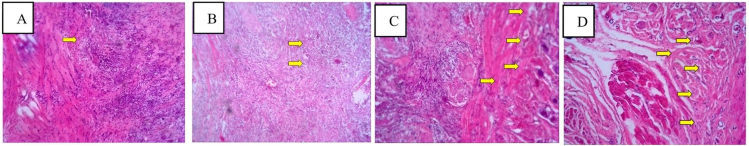
Histology of macrophages (yellow arrows) on day 4 for traumatic ulcers in oral mucosa of Wistar rats (HE; 100×). (A) Control group, (B) treatment group with 20% *Stichopus herrmanni* extract, (C) 40% extract treatment group, and (D) 80% extract treatment group.

The macrophage counts on day 4 are shown in Figure 4, and their histological appearance is illustrated in Figure 5. The highest macrophage count was observed in the 80% extract group, followed by the 40% and 20% extract groups, and the lowest in the control group. Statistical analyses detected significant differences between the control group and 80% extract group, as well as between the 20% and 80% extract groups (*p* < 0.05). No significant differences were found between the control group and the 20% or 40% extract groups (*p* > 0.05). According to histological observations, macrophages were identified as large cells with oval to kidney-shaped nuclei and abundant cytoplasm within the ulcer area (Figure 5). Fewer macrophages were scattered within the inflammatory infiltrate in the control group, whereas the macrophage distribution was denser in the 80% extract group. These findings indicate that the macrophage counts increased following administration of the highest concentration of *Stichopus herrmanni* extract.

### Angiogenesis

**Figure 6:**
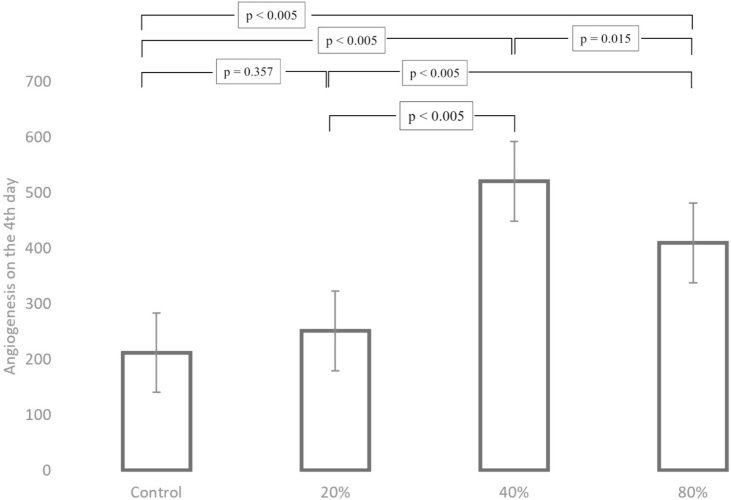
Effect of *Stichopus herrmanni* extract on angiogenesis counts on day 4.

**Figure 7:**
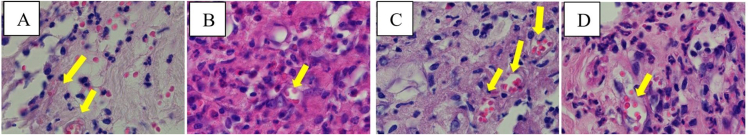
Histology of angiogenesis/blood vessels (yellow arrow) on day 4 for traumatic ulcers in oral mucosa of Wistar rats (HE; 100×). (A) Control group, (B) 20% *Stichopus herrmanni* extract treatment group, (C) 40% extract treatment group, and (D) 80% extract treatment group.

Quantitative analysis of angiogenesis on day 4 is presented in Figure 6, and representative histological findings are shown in Figure 7. The number of newly formed blood vessels was higher in all of the extract-treated groups compared with the control group, with the highest angiogenesis observed in the 40% extract group. Statistical analyses detected significant differences between the control group and 40% extract group, the control group and 80% extract group, the 20% and 40% extract groups (*p* < 0.05), and the 40% and 80% extract groups (*p* < 0.05). The histological observations in Figure 7 show newly formed capillaries characterized by round to oval lumens lined by endothelial cells, which were sparsely distributed in the control group and more densely arranged in the 40% and 80% extract groups. These results show that neovascular formation was enhanced following treatment with *Stichopus herrmanni* cucumber extract.

### Re-epithelialization

**Figure 8:**
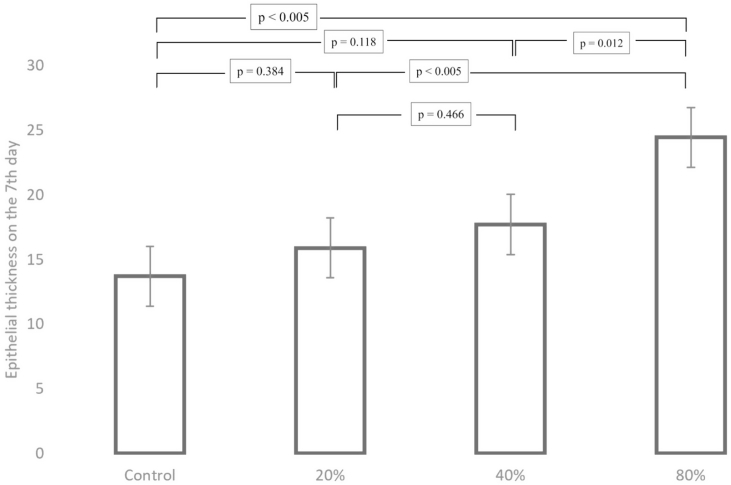
Effect of *Stichopus herrmanni* extract on epithelial thickness counts on day 7.

**Figure 9:**
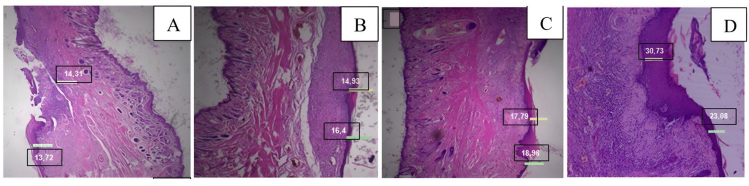
Histology of epithelium for traumatic ulcers in oral mucosa of Wistar rats (with HE staining) on day 7. (A) Control group, (B) 20% *Stichopus herrmanni* extract treatment group, (C) 40% extract treatment group, and (D) 80% extract treatment group.

The epithelial thickness on day 7 is shown in Figure 8, and the histological features are illustrated in Figure 9. The epithelial thickness was greatest in the 80% extract group, followed by the 40% and 20% extract groups, and the control group had the thinnest epithelium. Statistical analyses detected significant differences between the control group and 80% extract group, and between the 20% and 80% extract groups (*p* < 0.05). No significant differences were found between the control group and the 20% extract group, or between the 40% and 80% extract groups (*p* > 0.05). According to histological observations, Figure 9 shows that the epithelial layer covering the ulcer area was continuous and thicker in the 80% extract group, whereas the epithelium was thinner with less mature epithelial organization in the control group. These findings indicate that the epithelial thickness increased following treatment with the highest concentration of extract.

### Collagen

**Figure 10:**
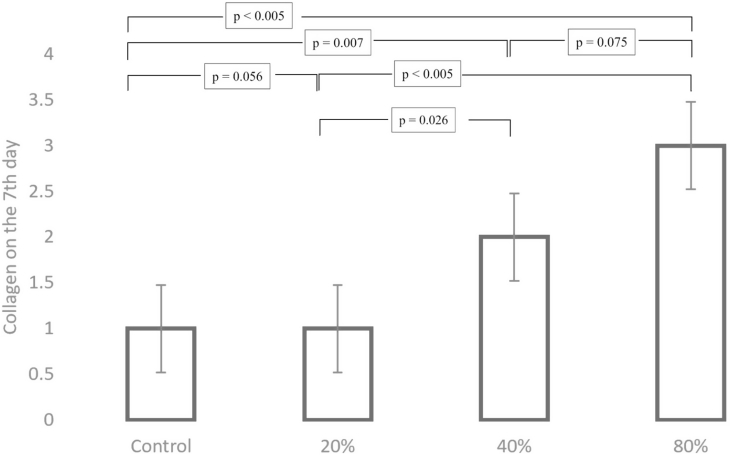
Effect of *Stichopus herrmanni* extract on collagen counts on day 7.

**Figure 11:**
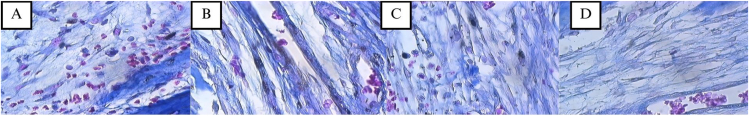
Histology of epithelium for traumatic ulcers in oral mucosa of Wistar rats (with Masson's trichrome staining) on day 7. (A) Control group, (B) 20% *Stichopus herrmanni* extract treatment group, (C) 40% extract treatment group, and (D) 80% extract treatment group.

The collagen density on day 7 is presented in Figure 10. The collagen density score was highest in the 80% extract group, whereas the collagen density values were similar for the 20% extract group and control group. Statistical analyses detected significant differences between the control group and 80% extract group, and between the 20% and 80% extract groups (*p* < 0.05). No significant difference was observed between the control group and 20% extract group (*p* > 0.05). As shown in Figure 11, histological evaluation using Masson's trichrome staining detected dense and well-organized collagen fibers in the 80% extract group, but thinner and more loosely arranged collagen fibers in both the control and 20% extract groups. These findings indicate that increased collagen deposition was associated with the highest concentration of *Stichopus herrmanni* extract.

## Discussion

*Stichopus herrmanni* is a marine-derived organism that has attracted considerable attention due to its rich contents of bioactive compounds, including collagen, glycosaminoglycans, chondroitin sulfate, and heparan sulfate, which are associated with tissue repair and anti-inflammatory activities.^4^ Previous characterization studies demonstrated that *Stichopus herrmanni* extract contains several bioactive compounds associated with wound healing activity, including proteins (19.39%), hyaluronic acid (3.98%), chondroitin sulfate (7.01%), dermatan sulfate (2.36%), and heparan sulfate (2.79%). These bioactive components are known to contribute to anti-inflammatory activity, angiogenesis, extracellular matrix remodeling, and collagen formation during the wound healing process.^8^

### Mast cells

The results obtained in the present study demonstrated that administration of *Stichopus herrmanni* extract at concentrations of 20%, 40%, and 80% increased the mast cell numbers on day 4, followed by a decrease on day 7. This pattern indicates a regulated inflammatory response, where mast cell recruitment occurs during the early inflammatory phase and subsequently declines as wound healing progresses toward the proliferative phase. By contrast, there was no significant difference in the mast cell numbers between days 4 and 7 in the control group, suggesting a prolonged inflammatory phase. The inflammatory phase of wound healing typically begins within 24 h after injury and lasts ∼4–6 days, involving neutrophils, monocytes, macrophages, mast cells, and T lymphocytes. The lower number of mast cells observed on day 7 in the treatment groups indicates an accelerated transition from inflammation to proliferation, and this effect was probably associated with the bioactive components of *Stichopus herrmanni*, particularly hyaluronic acid and minerals, which have anti-inflammatory and bacteriostatic properties. These components may modulate inflammatory mediators, thereby shortening the inflammatory phase.^9^ These findings suggest that the higher concentration *Stichopus herrmanni* extracts promoted faster resolution of inflammation in traumatic oral ulcers.

Mast cells play a crucial role in wound healing through the release of cytokines, chemokines, and growth factors that regulate inflammation, angiogenesis, and re-epithelialization. Mast cell recruitment to the wound site is stimulated by monocyte chemoattractant protein-1 (MCP-1) secreted by keratinocytes and macrophages. Upon activation, mast cells degranulate and release histamine, serotonin, tumor necrosis factor (TNF), proteases, and vasoactive mediators that increase vascular permeability and facilitate leukocyte infiltration. In addition, mast cells produce vascular endothelial growth factor (VEGF), interleukin (IL)-6, IL-8, basic fibroblast growth factor (bFGF), and platelet-derived growth factor (PDGF), which contribute to angiogenesis and fibroblast proliferation. Tryptase released from mast cells activates protease-activated receptor-2 (PAR-2) on endothelial cells, further promoting vasodilation.^10^ Mast cell-derived mediators such as FGF-2, VEGF, transforming growth factor-β (TGF-β), and nerve growth factor (NGF) also support fibrin formation and re-epithelialization.^7^ Therefore, the controlled increase and subsequent decrease in the number of mast cells observed in this study reflects an effective inflammatory response to support optimal wound healing.^8^^,^^9^

### Macrophages

The results obtained in this study showed that traumatic ulcers treated with *Stichopus herrmanni* extract had significantly higher numbers of macrophages compared with the control group. The macrophage count was highest in the 40% extract concentration, indicating that this concentration may be optimal for enhancing the inflammatory phase. Interestingly, increasing the concentration to 80% did not result in a further significant increase, suggesting a threshold effect. Macrophages are essential regulators of wound healing, and suppression of macrophage activity can delay tissue repair. During the inflammatory phase, macrophages phagocytose bacteria, necrotic tissue, and debris more efficiently than neutrophils. Macrophages also secrete proinflammatory cytokines such as IL-1, IL-6, and TNF-α, which are necessary for initiating tissue repair. Furthermore, macrophages serve as a bridge between the inflammatory and proliferative phases of wound healing. The increased macrophage count observed on day 4 in the treatment groups may have supported accelerated wound progression.^11^^,^^12^

Macrophages contribute to wound healing by producing growth factors such as VEGF, FGF, and TGF-β, which promote angiogenesis, fibroblast proliferation, and extracellular matrix formation. In addition, macrophages play an immunological role by processing and presenting antigens to lymphocytes, facilitating adaptive immune responses.^13^ The presence of hyaluronic acid in *Stichopus herrmanni* extract may enhance macrophage recruitment and activation. Hyaluronic acid fragments interact with toll-like receptors (TLR-2 and TLR-4) on macrophages and lymphocytes, stimulating the release of TNF-α, IL-6, IL-8, and IL-1.^14^^,^^15^ This interaction promotes a controlled inflammatory response and facilitates transition to the proliferative phase. The ability of *Stichopus herrmanni* extract to modulate macrophage activity probably contributed to the accelerated wound healing observed in the present study. These findings support a role for *Stichopus herrmanni* extract as an effective modulator of inflammation in traumatic oral ulcers.

The absence of macrophage evaluation on day 7 in the present study was based on the physiological pattern of acute wound healing, where macrophage activity predominantly occurs during the early inflammatory phase, particularly around days 3–4 after injury.^15^ During the transition to the proliferative phase, the inflammatory cell activity gradually decreases and is replaced by tissue repair processes such as collagen deposition and re-epithelialization. Persistent elevation of macrophages during later stages may indicate prolonged inflammation and delayed healing.^16^ Therefore, the observation of macrophages on day 4 was considered sufficient to represent the inflammatory response in this study.

### Angiogenesis

Angiogenesis was significantly enhanced in traumatic ulcers treated with *Stichopus herrmanni* extract compared with the control group, as evidenced by an increased number of newly formed blood vessels. The 40% extract concentration produced the highest angiogenic response, indicating optimal stimulation of neovascularization. Both the 40% and 80% extracts produced significantly greater angiogenesis than the control group, suggesting that the *Stichopus herrmanni* extract effectively promoted vascular formation. Angiogenesis is a critical component of the proliferative phase of wound healing, occurring between days 3 and 21 post-injury. Newly formed blood vessels supply oxygen and nutrients necessary for fibroblast proliferation, collagen synthesis, and epithelial regeneration. Initially, the wound area is avascular and depends on diffusion from surrounding capillaries. The formation of new capillary networks restores an adequate blood supply to the healing tissue.^17^

VEGF plays a central role in angiogenesis by promoting endothelial cell proliferation, migration, and increased vascular permeability. Components of *Stichopus herrmanni* extract, such as chondroitin sulfate and dermatan sulfate,^18^ stimulate macrophages to produce nitric oxide (NO), which enhances VEGF expression through hypoxia-inducible factor-1α (HIF-1α).^19^ During wound healing, the increased metabolic demand leads to localized hypoxia, further stimulating angiogenic signaling pathways. *Stichopus herrmanni* extract is rich in protein, collagen, minerals, glycosaminoglycans, and amino acids, including arginine. Arginine contributes to increased NO production, thereby enhancing VEGF-mediated angiogenesis.^20^ In addition, the heparin and heparan sulfate present in *Stichopus herrmanni* extract facilitate VEGF activation and endothelial cell migration. These mechanisms collectively explain the enhanced angiogenic response observed in this study.^21^

### Re-epithelialization

Re-epithelialization is a fundamental process in wound healing for restoring epithelial integrity following tissue injury. This process occurs during the proliferative phase and begins within 12–24 h after injury through mitotic activation of basal keratinocytes. After injury, keratinocytes undergo morphological changes, lose hemidesmosomal attachments to the basement membrane, and transition from a stationary to a migratory phenotype. Keratinocyte migration toward the wound bed is facilitated by the “free edge effect,” where cells at the wound margin actively move to cover the defect.^22^ Keratinocyte proliferation becomes more prominent within 48–72 h post-injury, supported by growth factors such as epidermal growth factor, TGF-α, and keratinocyte growth factor.^23^ As re-epithelialization progresses, the epithelial thickness increases and structural connections with the lamina propria are re-established. The formation of rete pegs around day 5 indicates effective epithelial anchoring and tissue maturation. By day 7, epithelial maturation is characterized by the development of a corneum-like layer.

In this study, the epithelial thickness was significantly greater on day 7 for traumatic ulcers treated with *Stichopus herrmanni* extract compared with the control group. The epithelial thickness was highest in the 80% extract group, followed by the 40% and 20% groups, indicating a dose-dependent effect. This enhanced epithelial response may be attributed to the high contents of glycosaminoglycans, including hyaluronic acid, chondroitin sulfate, and heparin, in *Stichopus herrmanni* extract.^24^ Hyaluronic acid plays a key role in the extracellular matrix by retaining water, facilitating keratinocyte migration, and stimulating cytokine production by macrophages. Hyaluronic acid promotes keratinocyte proliferation and migration through interacting with CD44 receptors. In addition, glycosaminoglycan-bound FGF enhances epithelial migration and wound contraction.^25^ These bioactive components create a favorable microenvironment for epithelial regeneration, leading to accelerated re-epithelialization.

### Collagen

In the present study, the collagen density was highest in the group treated with 80% *Stichopus herrmanni* extract, followed by the 40% and 20% groups, and the density was lowest in the control group. These findings indicate that higher concentrations of *Stichopus herrmanni* extract enhanced collagen synthesis during wound healing. Significant differences were observed between the 40% and 80% treatment groups compared with the control group, confirming the stimulatory effect of the extract on collagen deposition. Collagen plays a vital role in providing tensile strength and structural integrity to healing tissue. Fibroblasts are the primary cells responsible for collagen synthesis during the proliferative and remodeling phases. The increased collagen density observed in this study suggests that the fibroblast activity was enhanced in response to *Stichopus herrmanni* extract.^26^ Optimal collagen deposition is essential for successful wound closure and tissue remodeling.

*Stichopus herrmanni* extract contains glycosaminoglycans, such as chondroitin sulfate, dermatan sulfate, and heparin, which regulate fibroblast proliferation and collagen synthesis. Chondroitin sulfate binds to FGF-2, maintaining its availability in the tissue and promoting fibroblast proliferation.^27^ Heparin enhances collagen production and deposition, and dermatan sulfate supports fibroblast migration and adhesion. Proteoglycans regulate the activity of growth factors such as VEGF and FGF, which are involved in extracellular matrix formation. In addition, the zinc content of *Stichopus herrmanni* extract contributes to collagen synthesis and cross-linking. Collectively, these bioactive components explain the increased collagen density observed in the treatment groups.

This study had several limitations that should be considered when interpreting the findings. First, the relatively small sample size may limit the statistical power and generalizability of the results, although standardized experimental conditions and homogeneous animal characteristics were applied to minimize biological variability. Second, this study focused primarily on histopathological evaluation and did not include molecular analysis of inflammatory cytokines, growth factors, or signaling pathways involved in oral wound healing. Third, the observation period was limited to days 4 and 7, which may not have been fully representative of the complete remodeling phase of tissue healing. In addition, the present study was conducted using an experimental animal model, so the biological responses observed may differ from those in human oral mucosal wounds. Future studies involving longer observation periods, molecular evaluations, and larger experimental models are recommended to further clarify the therapeutic potential of *Stichopus herrmanni* extract in oral ulcer healing and the associated mechanisms.

## Conclusions

*Stichopus herrmanni* extract gel accelerated traumatic oral ulcer healing in Wistar rats by modulating inflammation, enhancing angiogenesis, promoting re-epithelialization, and increasing collagen deposition. The 40% concentration was more effective during the angiogenesis phase, whereas the 80% concentration had broader effects in both the inflammatory and proliferative phases, according to the increases in the macrophage count, epithelial thickness, and collagen density. These findings suggest that different concentrations of *Stichopus herrmanni* extract may influence different stages of oral wound healing. Therefore, *Stichopus herrmanni* extract has potential for use as a natural adjunctive therapy for traumatic oral ulcers.

## Ethical approval

Ethical approval for this study was obtained from the Health Research Ethics Committee of the Faculty of Dentistry, Universitas Airlangga, Surabaya, Indonesia, under approval number 1294/HRECC.FODM/XII/2025. All experimental procedures involving animals were conducted in accordance with institutional guidelines and relevant regulations for the care and use of laboratory animals.

## Authors contributions

All authors confirm that they made substantial contributions to the conception and design of the study, acquisition of data, and analysis and interpretation of data. All authors participated in drafting the article and revising it critically for important intellectual content. All authors approved the final version of the manuscript to be published and agree to be accountable for all aspects of the work. The authors confirm that the manuscript has been checked for plagiarism and that all individuals listed as authors meet the authorship criteria established by the journal.

## Source of funding

This study did not receive any specific grant from funding agencies in the public, commercial, or not-for-profit sectors. The research was conducted using the authors' personal funds.

## Conflict of interest

The authors have no conflict of interest to declare.

## References

[1] Sonar P.R., Panchbhai A., Kaur G., Jain M., Singh A., Thomas T. (2024). Chronic traumatic ulcer: a case report. Cureus.

[2] Zou Q.L., Tang Z.Q., Huang L.S., Wang X.H., Bao Z.X. (2024). Association between age, gender, and oral traumatic ulcerative lesions: a retrospective study. BMC Oral Health.

[3] Geahchan S., Baharlouei P., Rahman A. (2022). Marine collagen: a promising biomaterial for wound healing, skin anti-aging, and bone regeneration. Mar Drugs.

[4] Sim S.L., Kumari S., Kaur S., Khosrotehrani K. (2022). Macrophages in skin wounds: functions and therapeutic potential. Biomolecules.

[5] Chuhuaicura P., Rodríguez-Niklitschek C., Oporto G.H., Salazar L.A. (2025). Distinct molecular mechanisms in oral mucosal wound healing: translational insights and future directions. Int J Mol Sci.

[6] Pesterau A.M., Popescu A., Sirbu R., Cadar E., Busuricu F., Dragan A.L. (2025). Marine jellyfish collagen and other bioactive natural compounds from the sea, with significant potential for wound healing and repair materials. Mar Drugs.

[7] Zhang L., Nie F., Zhao J., Li S., Liu W., Guo H. (2024). PGRN is involved in macrophage M2 polarization regulation through TNFR2 in periodontitis. J Transl Med.

[8] Sari R.P., Budijono S.C.S. (2020). Characterization and potency of *Stichopus hermanni* ethanol extract on oral wound healing. Odonto Dent J.

[9] Murata M., Hara K., Saku T. (1997). Dynamic distribution of basic fibroblast growth factor during epulis formation: an immunohistochemical study in an enhanced healing process of the gingiva. J Oral Pathol Med.

[10] Lee J.H., Lee K.E., Kang S.W., Park S.H., Chae Y.K., Lee M.H. (2024). Effect of orodispersible hyaluronic acid film on palatal mucosa wound healing. Oral Dis.

[11] Cruz M.A., Araujo T.A., Avanzi I.R., Parisi J.R., de Andrade A.L.M., Rennó A.C.M. (2021). Collagen from marine sources and skin wound healing in animal experimental studies: a systematic review. Mar Biotechnol.

[12] Wang L.X., Zhang S.X., Wu H.J., Rong X.L., Guo J. (2019). M2b macrophage polarization and its roles in diseases. J Leukoc Biol.

[13] Liu L., Stokes J.V., Tan W., Pruett S.B. (2022). An optimized flow cytometry panel for classifying macrophage polarization. J Immunol Methods.

[14] Komi D.E.A., Khomtchouk K., Santa Maria PL. (2020). A review of the contribution of mast cells in wound healing: involved molecular and cellular mechanisms. Clin Rev Allergy Immunol.

[15] Cui Y., Hong S., Xia Y., Li X., He X., Hu X. (2023). Melatonin engineering M2 macrophage-derived exosomes mediate endoplasmic reticulum stress and immune reprogramming for periodontitis therapy. Adv Sci (Weinh).

[16] Joshi V.M., Kandaswamy E., Germain J.S., Schiavo J.H., Fm H.S. (2024). Effect of hyaluronic acid on palatal wound healing: a systematic review. Clin Oral Invest.

[17] Keswani S.G., Balaji S., Le L.D., Leung A., Parvadia J.K., Frischer J. (2013). Role of salivary vascular endothelial growth factor in palatal mucosal wound healing. Wound Repair Regen.

[18] Plichta J.K., Radek K.A. (2012). Sugar-coating wound repair: a review of FGF-10 and dermatan sulfate in wound healing and their potential application in burn wounds. J Burn Care Res.

[19] Mathew-Steiner S.S., Roy S., Sen C.K. (2021). Collagen in wound healing. Bioengineering (Basel).

[20] Qiang L., Yang S., Cui Y.H., He Y.Y. (2021). Keratinocyte autophagy enables the activation of keratinocytes and fibroblasts and facilitates wound healing. Autophagy.

[21] Selnø A.T.H., Schlichtner S., Yasinska I.M., Sakhnevych S.S., Fiedler W., Wellbrock J. (2020). Transforming growth factor beta type 1 (TGF-β) and hypoxia-inducible factor 1 (HIF-1) transcription complex as master regulators of the immunosuppressive protein galectin-9 expression in human cancer and embryonic cells. Aging (Albany NY).

[22] Pramanik A., Xu Z., Ingram N., Coletta P.L., Millner P.A., Tyler A.I.I. (2022). Hyaluronic-acid-tagged cubosomes deliver cytotoxics specifically to CD44-positive cancer cells. Mol Pharm.

[23] Ground M., Waqanivavalagi S., Park Y.E., Callon K., Walker R., Milsom P. (2022). Fibroblast growth factor 2 inhibits myofibroblastic activation of valvular interstitial cells. PLoS One.

[24] Chew E.Y., Clemons T.E., Agrón E., Domalpally A., Keenan T.D.L., Vitale S. (2022). Long-term outcomes of adding lutein/zeaxanthin and ω-3 fatty acids to the AREDS supplements on age-related macular degeneration progression: AREDS2 report 28. JAMA Ophthalmol.

[25] Gao Q., Shang Y., Zhou W., Deng S., Peng C. (2022). Marine collagen peptides: a novel biomaterial for the healing of oral mucosal ulcers. Dent Mater J.

[26] Hutami I.R., Izawa T., Khurel-Ochir T., Sakamaki T., Iwasa A., Tanaka E. (2021). Macrophage motility in wound healing is regulated by HIF-1α via S1P signaling. Int J Mol Sci.

[27] Shi Z., Yao C., Shui Y., Li S., Yan H. (2023). Research progress on the mechanism of angiogenesis in wound repair and regeneration. Front Physiol.

